# Diet quality and dietary acid load in relation to cardiovascular disease mortality: Results from Fasa PERSIAN cohort study

**DOI:** 10.1002/fsn3.3197

**Published:** 2022-12-23

**Authors:** Sahar Fereidouni, Najmeh Hejazi, Reza Homayounfar, Mojtaba Farjam

**Affiliations:** ^1^ Student Research Committee, School of Nutrition and Food Sciences Shiraz University of Medical Sciences Shiraz Iran; ^2^ Nutrition Research Center, Department of Clinical Nutrition, School of Nutrition and Food Sciences Shiraz University of Medical Sciences Shiraz Iran; ^3^ National Nutrition and Food Technology Research Institute, Faculty of Nutrition Sciences and Food Technology Shahid Beheshti University of Medical Sciences Tehran Iran; ^4^ Noncommunicable Diseases Research Center Fasa University of Medical Sciences Fasa Iran

**Keywords:** alternative healthy eating index, CVD mortality, DASH diet, dietary acid load, dietary inflammatory index, Mediterranean diet

## Abstract

Dietary intake is a determining factor in the morbidity and mortality of chronic disorders. However, not many documents have investigated this relationship. The aim of this study was to evaluate the associations of the Mediterranean dietary score (MDS), Alternative Healthy Eating Index (AHEI), Dietary Inflammatory Index (DII), DASH score, and dietary acid load with cardiovascular disease (CVD) mortality. A total of 2158 CVD patients (mean age of 54.73 ± 8.62 years) from the Fasa cohort study, Iran, participated in the current study. *Diet quality indices including* DII, AHEI, MDS, DASH, and dietary acid load (NEAP score) were computed using a validated 125‐item Food Frequency Questionnaire (FFQ). Cox regression analyses were used to determine HRs and 95% CIs. During a follow‐up of 3 years, we documented 59 CVD deaths. After adjusting for relevant confounders (age, gender, family history of CVD, smoking, physical activity, alcohol intake, and HTN) in the final model, we found that higher DII scores and dietary acid load were significantly related to increased mortality due to CVD (HR = 1.11; 95% CI = 1.01–1.24; and HR = 1.02; 95% CI = 1.01–1.03). However, the DASH score was insignificantly associated with decreased CVD mortality by 20.4% (HR = 0.79; 95% CI = 0.57–1.09). There was no significant relationship among AHEI score, MDS, and CVD mortality. This study showed that increasing dietary acidity and the use of inflammatory food compounds could contribute to CVD mortality. Also, adherence to the DASH diet may be associated with reduced CVD mortality.

## Graphical abstract

The aim of this study was to evaluate the associations of the Mediterranean dietary score (MDS), Alternative Healthy Eating Index (AHEI), Dietary Inflammatory Index (DII), DASH score, and dietary acid load with cardiovascular disease (CVD) mortality. This study showed that increasing dietary acidity and the use of inflammatory food compounds could contribute to CVD mortality. Also, adherence to the DASH diet may be associated with reduced CVD mortality.

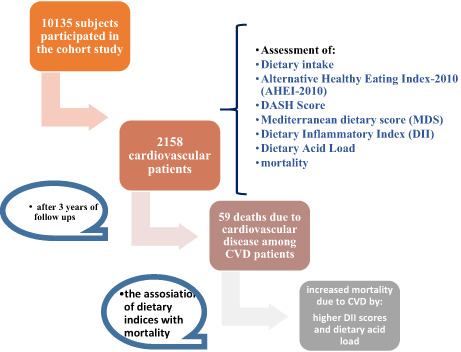

## 1 INTRODUCTION

Cardiovascular disease (CVD) is referred to as a group of related disorders that include ischemic heart disease, hypertension, atherosclerosis, heart failure, and *peripheral arterial disease*. The prevalence of CVD and its mortality have been 422.7 million cases and 17.9 million, during a year in the world, respectively (Emamian et al., 2020; Sarrafzadegan & Mohammmadifard, 2019). Evidence has shown that age, genetics, ethnicity, gender, hypertension, smoking history, abdominal obesity, physical inactivity, and poor diet are the risk factors for CVD; nevertheless, by identifying these risk factors in individuals, we can adopt an appropriate strategy for treatment and prevention (Sarrafzadegan & Mohammmadifard, 2019). Among the risk factors, dietary modification was an important approach for the management and prevention of CVD and its mortality. A diet with excessive sodium, saturated fatty acids, cholesterol, refined grains, red and processed meat, and alcohol could increase the risk of CVD and its mortality, while consumption of fruits, seafood, vegetables, whole grains legumes, nuts, and unsaturated fatty acids is shown to be linked to reduced mortality of CVD (Aigner et al., 2018). Evidence suggested that Mediterranean dietary patterns and the dietary approach to stop hypertension (DASH) diet could reverse the CVD progression (Levitan et al., 2013). Previous documents for the control and management of CVD focused mainly on single micronutrients or food items. However, these days, instead of evaluating single nutrients or groups of food, analysis of dietary patterns and dietary indices considers interactions and dependencies among nutrients and provides appropriate strategies for chronic disease management and prevention (Aigner et al., 2018).

The quality of the diet can be assessed by the Alternative Healthy Eating Index (AHEI), Mediterranean diet score (MED), and DASH scores indices. According to a meta‐analysis, the higher the score of these indices was significantly inversely associated with CVD progression and all‐reason mortality (Aigner et al., 2018; Akbaraly et al., 2011).

Recently, studies introduce acid–base dietary imbalance as a risk factor for chronic diseases, so a higher dietary acid load increases the incidence of CVD, hypertension, and related mortality (Akter et al., 2017). Consuming low amounts of vegetables and fruits simultaneously with eating excessive processed red meat can reduce the quality of the diet and increase its acid load (Fatahi & Azadbakht, 2019). The dietary acid load could be elevated using dietary acid load (DAL), potential renal acid load (PRAL), and net endogenous acid production (NEAP). Higher scores of PRAL, DAL, and NEAP scores show more acid‐loading potential (Fatahi & Azadbakht, 2019).

Evidence has demonstrated that inflammation is involved in the stages of atherosclerosis leading to plaque rupture and thrombosis which increase the probability of CVD mortality. In this regard, a diet rich in antiinflammatory ingredients plays an important role in reducing the severity and consequence of inflammatory chronic diseases the same as CVDs and this mortality. For evaluating the antiinflammatory and inflammatory strength of a diet, a dietary inflammatory index (DII) is recommended. A higher score of DII, indicating a higher inflammatory potential of diet, has been related to the risk of CVD and its mortality (Hodge et al., 2018; Shivappa et al., 2014).

The recent study aimed to investigate the prospective relationships of the DII, DASH, MDS, AHEI score, and dietary acid load with the mortality of CVD in the context of the epidemiologic Persian cohort study.

## 2 METHODS

### 2.1 Study population

The present study included 1622 women and 536 men with cardiovascular diseases who participated in the Fasa PERSIAN cohort as a branch of the Prospective Epidemiological Research Study in Iran. It was conducted between November 2014 and June 2019. In this cohort study, 10,135 subjects aged 35 to 70 years who were not physically or mentally disabled, and lived in Sheshdeh, a district of Fasa, participated for more than 9 months each year (Farjam et al., 2016). A total of 2222 subjects were excluded from the study due to incomplete data on the intake of diet and mortality status as well as reports of abnormal energy intake (less than 800 kcal and more than 4200 kcal). Finally, a total of 2158 participants with CVD took part in the current research. Information on demographic, behavioral, anthropometric data, medical history, and intake of foods was assessed through questionnaires biennially in the cohort study; likewise, mortality status and the cause of mortality were determined during the annual follow‐up (Farjam et al., 2016). In this study, all procedures involving patients were approved by the Ethics Committee of Shiraz University of Medical Sciences, Shiraz, Iran (code: IR.SUMS.REC.1399.1116). Written informed consent was provided by all patients.

### 2.2 Dietary intake assessment and indices

The usual food intake of patients was determined using a validated 125‐item Food Frequency Questionnaire (FFQ) which was modified by the Iranian food culture (Farjam et al., 2016; Willett et al., 1985). An expert nutritionist in a face‐to‐face interview registered the amount of food consumption in the last year at the baseline of the cohort study. Nutritionist IV software (version 7.0) was used to determine the nutrient contents and energy of foods (Farjam et al., 2016).

### 2.3 Alternative healthy eating index‐2010 (AHEI‐2010)

Kennedy et al. designed the AHEI index to assess the quality of the diet (Schwingshackl, Bogensberger & Hoffmann, 2018). This index includes 11 components and the individuals are scored based on the consumption of these items. Legumes, vegetables, whole grains, nuts and fruits, DHA and EPA, and polyunsaturated fatty acids are positive components, while transfatty acids, sweetened beverages, sodium, and red and processed meats are the negative components; alcohol is the moderate component. For calculating, first, we categorized the individuals based on consumption deciles and then inverted the negative component decile score, and finally, summed the total score. The overall score was between 9 and 81. A higher AHEI score indicates a healthier diet (McCullough et al., 2002).

### 2.4 DASH score

There are different methods for calculating the DASH diet score. In this study, Mellen's DASH Index was applied. Its components included fiber, calcium, magnesium, cholesterol, sodium, potassium, protein, total fat, and saturated fat. The score was considered 1 point for the goal consumption and 0.5 for the intermediate intakes. The overall score ranged from 0 to 9. A higher score indicates greater adherence to the DASH diet (Miller et al, 2013).

### 2.5 Mediterranean dietary score (MDS)

In this study, we used MEDI‐LITE scoring for this pattern, which was described by Francesco Sophie et al. This score focuses on nine components (vegetable, dairy products, fruit, fish, meat, grains, meat products, legumes, olive oil, and alcohol). Higher intake of cereals, legumes, fruit, fish, and vegetables is scored 2 points, intermediate consumption 1 points, and lower intake of them 0 points. The scoring of dairy products, processed meat, and meat was the opposite of the previous groups. Medium consumption of alcohol was scored 2 points, the lowest consumption 1 point, and the highest intake 0 points. The final score was between 0 and 18 points. Higher scores indicate more adherence to the MDS (Sofi, Macchi, Abbate, Gensini & Casini, 2014).

### 2.6 Dietary inflammatory index (DII)

We computed the DII score based on 30 groups of food including energy, fat, transfat, cholesterol, carbohydrate, protein, saturated fat, vitamin B12, iron, MUFA, PUFA, vitamin D, vitamin B6, fiber, vitamin B9, vitamin C, niacin, thiamin, riboflavin, vitamin A, magnesium, vitamin E, β‐carotene, onion, garlic, tea, caffeine, selenium, and zinc. Consumption of groups such as eugenol, turmeric, saffron, ginger, pepper, rosemary, polyphenols, and anthocyanin was not available for the calculation of this index. First, the energy intake of the participants was adjusted based on 1000 kcal. Then, to calculate DII, we subtracted the dietary parameters from global average and divided it by the “global standard deviation” to get a Z score. The Z‐score values were then converted into percentiles. The percentile values were then multiplied by 2 minus 1. Finally, the scores obtained from each of the 30 parameters were multiplied by the overall inflammatory score; then, we summed up all food items to calculate the total DII score (Shivappa et al., 2014).

### 2.7 Dietary acid load

We constructed the acid load of the diet based on the consumption of several nutrients using three different methods: PRAL (mEq/d) = (0.49 × protein [g/d]) + (0.037 × Phosphorous [mg/d]) – (0.021 × potassium [mg/d]) – (0.013 × calcium [mg/d]) – (0.026 × magnesium [mg/d]), NEAP (mEq/day) = (protein [g/d] × 54.5/potassium [mEq/d])‐10.2, and DAL (mEq/day) = (body surface area [m^2^] × 41 [mEq/day]/1.73 m^2^) + PRAL. Du Bois formula: height^0.725^ × weight^0.425^ × 0.00718 was used for computing the surface of the body. Acid load score obtained from these three methods was used for statistical analysis (Han et al., 2016).

### 2.8 Assessment of other variables

Cardiovascular patients were followed up clinically during a 3‐year period. They were identified based on previous medical history, electrocardiography, laboratory sampling, validated screening questionnaires, and physical exams. Physical activity measurement was done by the International Physical Activity Questionnaire (IPAQ). Using a digital scale (Tanita BC‐418, Tanita Corp, Japan), we recorded the weight and height. Body mass index (BMI) was then computed by weight (kg)/height (m)^2^. Waist circumference was measured with a precision of 0.1 cm. The medical history and lifestyle factors that are associated with CVD risk were recorded (Farjam et al., 2016).

### 2.9 Outcome assessment

In this study, our outcome was cardiovascular mortality; based on the 10th edition of the International Classification of Diseases (ICD), deaths due to the following diseases are considered CVD mortality: hypertension (ICD I10‐I15), peripheral vascular disease (ICD I70‐I89), ischemic heart disease (ICD I20‐I25), stroke (ICD I60‐I69), and pulmonary heart disease (ICD I26‐I28). The patients' CVD mortality was followed for 3 years after the first year of enrollment in the study.

### 2.10 Statistical analysis

SPSS software version 21 was used for data analysis. Quantitative variables are reported as mean ± standard error and qualitative data are presented as frequency (percentage). The normality of the distribution of variables was assessed using the Kolmogorov–Smirnov test. Chi‐Square tests and Mann–Whitney U tests were used to compare qualitative and quantitative data among the participants based on gender, respectively. A Cox regression analysis was used to assess the relationship among the four dietary quality indices (DII, DASH score, MDS, and AHEI), dietary acid load, and CVD mortality. The outcome was defined as mortality, the temporal factor was time to the event, and exposure was one of the dietary indices. We did not adjust for any covariates in the first model (basic model). The second model was adjusted for covariates including age, gender, smoking, level of physical activity, alcohol intake, family history of CVD, and history of HTN. The third model was adjusted by total energy, waist‐to‐hip ratio (WHR), weight, BMI, DBP, SBP, TG, cholesterol, LDL, and HDL. Finally, the model was adjusted with all the components of models 2 and 3. The hazard ratio (AR) and 95% confidence interval (CI) were presented to show the strength of the relationship between dietary quality indices including DII, AHEI, MDS, and DASH score, and dietary acid load and CVD mortality. *p*‐values of <.05 were considered significant.

## 3 RESULTS

A total of 2158 individuals (24.8% males and 75.2% females), with a mean age of 54.73 ± 8.62 years, participated in the current study. During a 3‐year follow‐up of the present study participants, 59 deaths (30 males and 29 females) due to CVD got recorded.

Table 1 displays the basic characteristics of the study patients. As shown in Table 1, the mean weight, age, and serum cholesterol were significantly higher in men with CVDs compared to women (*p* = <.05), but their BMI, WHR, and the serum level of TG, HDL, and LDL were significantly lower (*p* = <.05). Based on Table 1, the frequency distribution of the participants in terms of the history of hypertension was significantly higher in women with CVDs compared to men. (*p* = <.05). However, the frequency distribution of the records of alcohol consumption and active smoking was significantly lower (*p* = <.05). Table 1 also shows that 5.6 and 1.8 percent of men and women with CVDs died due to cardiovascular events, which is significantly higher in men (*p* = <.05) (Table 1).

**TABLE 1:** Distribution of baseline variables in men and women

	Total	Men (*n* = 536)	Women (*n* = 1622)	*p*‐value
Age (year)^a^	54.73 ± 8.62	57.04 ± 8.81	54.41 ± 9.02	<.0001
Weight (Kg)^a^	67.76 ± 1.32	71.13 ± 13.47	66.67 ± 12.83	<.0001
BMI (kg/m^2^)^a^	27.09 ± 4.94	25.30 ± 4.28	27.7 ± 4.96	<.0001
WHR^a^	0.96 ± 0.06	0.94 ± 0.06	0.96 ± 0.06	<.0001
Physical activity (MET)^a^	38.45 ± 8.38	40.68 ± 12.47	37.7 ± 6.3	.65
DBP (mmHg)^a^	79.97 ± 13	79.65 ± 13.83	80.07 ± 12.73	.63
SBP (mmHg)^a^	123.04 ± 21.64	122.67 ± 22.28	123.1473 ± 21.44	.82
Daily energy intake (kcal)^a^	2670.32 ± 750.17	2985.86 ± 693.26	2564.09 ± 739.32	<.0001
History of HTN (%)^b^	79.1	64.6	84.0	<.0001
alcohol consumption (%)^b^	0.9	3.5	0.0	<.0001
Active smoking (%)^b^	18.6	50.4	8.1	<.0001
FBS	102.9 ± 41.44	101.91 ± 37.50	103.32 ± 42.73	.96
Cholesterol (mg/dl)^a^	189.18 ± 43.27	176.13 ± 45.58	193.47 ± 41.59	<.0001
TG (mg/dl)^a^	142.13 ± 86.10	139.57 ± 97.57	143.11 ± 82.08	.01
LDL (mg/dl)^a^	108.86 ± 36.05	101.50 ± 37.52	111.22 ± 35.19	<.0001
HDL (mg/dl)^a^	81.85 ± 15.49	46.71 ± 14.29	53.57 ± 15.53	<.0001
CVD mortality (%)^b^	3.1	5.6	1.8	<.0001

Abbreviations: BMI—body mass index, CVD—cardiovascular disease, DBP—diastolic blood pressure, FBS—fast blood sugar, HDL—high‐density lipoprotein, LDL—low‐density lipoprotein, SBP—systolic blood pressure, TG—triglyceride, WHR—waist‐to‐hip ratio.

*Note*: Values are presented as mean ± (standard deviation) or as % of patients.

^a^ Mann–Whitney U tests.

^b^ Chi‐square tests.

Dietary indices and intake of the participants of the study are reported in Table 2. As Table 2 indicates, the level of energy intake and macro‐ and micronutrients are significantly higher in men compared to women (*p* = <.05). The Table 2 did not show any significant difference in dietary indices among male and female participants of the study (Table 2).

**TABLE 2:** Distribution of the level of energy intake, macro‐ and micronutrients, mean score of dietary indices, and dietary acid load in men and women

	Total	Men (*n* = 536)	Women (*n* = 1622)	*p*‐value^a^
Daily energy intake (kcal)^a^	3667.97 ± 16.16	2985.86 ± 693.26	2564.09 ± 739.32	<.0001
Carbohydrate (gr)	465.01 ± 2.97	521.35 ± 5.61	446.40 ± 3.37	<.0001
Protein (gr)	81.54 ± 0.55	91.81 ± 1.08	78.15 ± 0.62	<.0001
Fat (gr)	58.04 ± 0.51	63.78 ± 1.10	56.14 ± 0.57	<.0001
Monounsaturated fatty acid (gr)	16.98 ± 0.17	19.15 ± 0.41	16.26 ± 0.18	<.0001
Polyunsaturated fatty acid (gr)	8.21 ± 0.08	9.12 ± 0.17	7.91 ± 0.09	<.0001
Saturated fatty acid (gr)	22.09 ± 0.26	23.53 ± 0.55	21.62 ± 0.30	.001
Alpha‐linolenic (gr)	0.33 ± 0.005	0.35 ± 0.011	0.32 ± 0.006	.008
Linoleic (gr)	0.13 ± 0.002	0.35 ± 0.01	0.32 ± 0.006	.008
Cholesterol (mg)	205.52 ± 2.50	250.47 ± 5.60	190.67 ± 2.67	<.0001
DHA (gr)	0.03 ± 0.0005	0.04 ± 0.001	0.02 ± 0.0005	<.0001
EPA (gr)	0.012 ± 0.0003	0.015 ± 0.0007	0.011 ± 0.0004	<.0001
Sodium (mg)	1281.49 ± 4.13	1316.18 ± 2.12	1356 ± 2.42	<.0001
Potassium (mg)	1157.35 ± 4.41	1212.12 ± 2.45	1102 ± 2.87	<.0001
Fiber (gr)	27.06 ± 0.23	28.56 ± 0.46	26.56 ± 0.26	<.0001
Calcium (mg)	513.88 ± 5.74	549.15 ± 10.89	502.22 ± 6.71	<.0001
Vitamin D (IU)	36.24 ± 0.61	48.89 ± 1.43	32.05 ± 0.63	<.0001
Vitamin C (mg)	141.47 ± 2.08	146.08 ± 3.78	139.95 ± 2.47	.039
Vitamin E (mg)	36.24 ± 0.61	32.05 ± 0.63	12.56 ± 0.23	<.0001
Vitamin K (Ug)	239.89 ± 5.91	235.13 ± 9.53	241.46 ± 7.21	.510
Folate (mg)	374.92 ± 4.73	403.90 ± 9.48	365.35 ± 5.43	<.0001
Iron (mg)	17.61 ± 0.12	19.26 ± 0.24	17.06 ± 0.14	<.0001
Zinc (mg)	6.40 ± 0.05	7.24 ± 0.11	6.12 ± 0.059	<.0001
Magnesium (mg)	259.82 ± 2.26	288.42 ± 4.48	250.37 ± 2.57	<.0001
Selenium (mg)	41.52 ± 0.47	49.89 ± 1.06	38.75 ± 0.49	<.0001
AHEI score^a^	62.05 ± 10.41	62.81 ± 10.96	61.79 ± 10.20	.06
MDS score^a^	9.12 ± 2.42	9.09 ± 2.45	9.14 ± 2.42	.47
DASH score^a^	3.45 ± 0.80	3.45 ± 0.82	3.44 ± 0.79	.70
DII score^a^	0.05 ± 2.62	−0.06 ± 2.44	0.08 ± 2.67	.14
NEAP^a^	49.42 ± 22.10	50.57 ± 24.88	49.00 ± 21.00	.58

Abbreviations: AHEI—Alternative Healthy Eating Index, DHA—docosahexaenoic acid, DII—Dietary Inflammatory Index, EPA—eicosapentaenoic acid, NEAP—net endogenous acid production.

^a^ Mann–Whitney U tests.

The association between different variables and CVDs mortality in a Cox univariate model is shown in Table 3. It was revealed that being older (HR = 1.065; 95% CI = 1.02–1.1; *p* = .001), having a history of HTN (HR = 4.62; 95% CI = 2.69–7.92; *p* = <.0001), being an active smoker (HR = 1.78; 95% CI = 1.02–3.11; *p* = .041), and being a man (HR = 3.46; 95% CI = 2.12–5.64; *p* = <.0001) were significantly associated with the increased risk of CVDs mortality. Also, there was an inverse significant relationship between the serum HDL level and CVDs mortality (HR = 0.97; 95% CI = 0.95–0.99; *p* = .02) (Table 3).

**TABLE 3:** The association between the variables and CVD mortality by Cox regression analysis relationship in the whole

Variable	*β*	*p*‐value	Hazard ratio (95% CI)
Age (year)	0.063	.001	1.065 (1.02–1.1)
Gender	1.24	<.0001	3.46 (2.12–5.64)
Weight (Kg)	0.030	.129	1.030 (0.99–1.07)
BMI (kg/m^2^)	−0.084	.16	0.91 (0.81–1.034)
WHR	0.364	.88	1.44 (0.01–1.97)
Physical activity (MET)	−0.027	.17	0.12 (0.94–1.007)
DBP (mmHg)	−0.026	.109	0.974(0.943–1.00)
SBP (mmHg)	0.008	.409	1.008(0.98–1.02)
Daily energy intake (kcal)	0.00	.24	1.00 (1.00–1.001)
History of HTN (%)	1.53	<.0001	4.62 (2.69–7.92)
alcohol consumption (%)	0.28	.78	1.32 (0.18–3.71)
Active smoking (%)	0.58	.041	1.78 (1.02–3.11)
FBS	0.007	<.0001	1.00 (1.00–1.01
Cholesterol (mg/dl)	0.005	.241	1.00 (0.99–1.01)
TG (mg/dl)	0.009	.95	1.00 (0.75–1.35)
LDL (mg/dl)	0.04	.95	1.04 (0.23–4.59)
HDL (mg/dl)	−0.02	.02	0.97 (0.95–0.99)

Abbreviations: BMI—body mass index, DBP—diastolic blood pressure, FBS—fast blood sugar, HDL—high‐density lipoprotein, LDL—low‐density lipoprotein, SBP—systolic blood pressure, TG—triglyceride, WHR—waist‐to‐hip ratio.

Table 4 demonstrates the associations among baseline dietary acid load, different dietary indices scores (DII, AHEI, MDS, and DASH scores), and CVDs mortality. As shown in Table 4, there was no significant relationship between dietary indices and CVDs mortality in the main univariate Cox regression models. The confidence interval included 1.00 for NEAP score. However, the fully adjusted model (model 4) reports that there was a significant positive relationship between the baseline DII score and dietary acid load (NEAP score) level with CVDs mortality (HR = 1.11; 95% CI = 1.01–1.24; *p* = .04 and HR = 1.02; 95% CI = 1.01–1.03; *p* = <.0001, respectively). We did not observe any correlation among AHEI, MDS scores, and CVD mortality (Table 4).

**TABLE 4:** The association among diet quality indices, dietary acid load, and CVD mortality by Cox regression analysis relationship in the whole

	*β*	*p*‐value	Hazard ratio (95% CI)
Model 1			
AHEI score	0.00	.80	1.03 (0.97–1.02)
MED score	0.04	.42	1.04 (0.93–1.16)
DASH score	0.24	.12	0.78 (0.57–1.07)
DII score	0.07	.15	1.07 (0.97–1.19)
NEAP	0.01	<.0001	1.01 (1.00–1.02)
Model 2			
AHEI score	0.006	.667	1.00 (0.98–1.03)
MED score	0.054	.313	1.05 (0.95–1.17)
DASH score	−0.233	.15	0.79 (0.57–1.08)
DII score	0.08	.122	1.083 (0.97–1.19)
NEAP	0.01	.001	1.01 (1.00–1.01)
Model 3			
AHEI score	0.008	.527	1.00 (0.98–1.03)
MED score	0.046	.396	1.04 (0.94–1.16)
DASH score	−0.274	.09	0.76 (0.55–1.04)
DII score	0.115	.035	1.12 (1.00–1.24)
NEAP	0.013	<.0001	1.01 (1.00–1.02)
Model 4			
AHEI score	0.006	.65	1.00 (0.98–1.03)
MED score	0.07	.19	1.07 (0.96–1.20)
DASH score	−0.22	.16	0.79 (0.57–1.09)
DII score	0.12	.047	1.11 (1.01–1.24)
NEAP	0.02	<.0001	1.02 (1.01–1.03)

Abbreviations: AHEI—Alternative Healthy Eating Index, DII—Dietary Inflammatory Index, NEAP—net endogenous acid production.

*Note*: Model 1: Not Adjusted; Model 2: Adjusted for age, gender, smoking, alcohol intake, level of physical activity, family history of CVD, and history of HTN; Model 3: Adjusted for total energy, waist‐to‐hip ratio (WHR), BMI, weight, DBP, SBP, TG, cholesterol, LDL, and HDL; Model 4: Adjusted for age, gender, smoking, alcohol intake, level of physical activity, family history of CVD, history of HTN, total energy, waist‐to‐hip ratio (WHR), BMI, weight, DBP, SBP, TG, cholesterol, LDL and HDL.

## 4 DISCUSSION

This retrospective cohort study was conducted to investigate the association between dietary indices and dietary acid load with CVD mortality in cardiovascular patients of Fasa PERSIAN cohort. The results of the current study showed that the CVD mortality rate increased significantly with age, being a man, and simultaneous onset of hypertension; there was also a significant positive relationship between tobacco use and mortality from cardiovascular diseases. However, it was found that with increasing serum HDL levels, the mortality rate of CVD decreased significantly. In addition, there was a direct and significant association between DII score and dietary acid load with CVD mortality; thus, with an increase in DII score and dietary acid load, the rate of CVD mortality increased by 11% and 2%, respectively, while there was no significant correlation between AHEI and MDS scores with CVD mortality. The current study also revealed that adherence to the DASH diet in cardiovascular patients can decrease the risk of CVD mortality by 20.4%, but this decrease was not statistically significant although it is clinically significant.

In line with our study, *Jibin* et al. (Tan et al., 2018) linked tobacco use and hypertension levels to mortality due to heart disease, stroke, and IHD. Nicotine and carbon monoxide available in tobacco promote the development of atherosclerosis by affecting myocardial oxygen capacity and increasing endothelial damage. Previous studies have also shown that tobacco use is closely linked to high hypertension and stroke. In addition, one of the main risk factors for heart disease is hypertension, and the severity of this disease can be controlled by reducing it (Tan et al., 2018; Wei et al., 1996).

This study also showed an inverse relationship between serum HDL levels and CVD mortality rates in patients. In this regard, *Chantal* et al. (Kopecky et al., 2015) reported high levels of serum HDL as a protective factor against mortality and cardiovascular diseases in persons with diabetes. HDL plays a protective role against cardiovascular diseases and their mortality by clearing cholesterol from the macrophages and increasing endothelial function and antioxidant activities (Kopecky et al., 2015).

Mikkola et al. (2013) in their study, like the present study, showed that the risk of CVD mortality was higher in men compared to women. In this study, we observed higher rate of smoking and alcohol consumption along with lower levels of HDL in men compared to women, which are all risk factors for CVD and the resulting mortality. In addition, higher levels of estrogen in women compared to men is a protective factor against cardiovascular disorders (Mikkola et al., 2013).

Similar to our study, (Hodge et al., 2018; Shivappa et al., 2014) observed a direct association between CVD mortality and DII score. To explain this association, we can point to the relationship between high DII scores and increased risk of obesity, metabolic syndrome, and insulin resistance.(Garcia‐Arellano et al., 2015; Hébert et al., 2014; Ramallal et al., 2017; Shivappa et al., 2014). These chronic diseases are associated with inflammatory conditions in the body. Inflammatory biomarkers including hs‐CRP, IL‐6, IL‐4, IL‐1B, IL‐10, and TNF‐α have also been shown to be connected with obesity, diabetes, and CVD. In this regard, previous studies have linked increased DII scores to high levels of cytokines such as IL‐1, TNF‐α, and CRP. On the other hand, the guidelines of AHA published in 2019 have introduced obesity and diabetes as two risk factors related to CVD mortality (Arnett et al., 2019; Choi et al., 2013; Shivappa et al., 2014).

According to the AHA‐2019 guideline, energy, saturated fatty acids, cholesterol, transfatty acids, red meat, and refined grains in the diet not only increased the DII score but are also linked to increased mortality due to CVDs (Arnett et al., 2019). In contrast, the antiinflammatory compounds such as vitamin C, zinc, vitamin E, and beta‐carotene are associated with a decrease in DII score, and the antioxidant role of these compounds is a factor in the primary and secondary prevention of cardiovascular diseases (Hodge et al., 2018). In this regard, omega‐3 fatty acids and polyphenols (found in vegetables and fruits) are associated with a decrease in DII score and inflammation rate in the body. They are involved in regulating the body's inflammatory processes, improving lipid profile, oxidative stress, and endothelial function, and in this way, decreasing the risk of chronic disorders such as CVD disease. As a result, if dietary intake leads to inflammatory conditions, it can increase the risk of platelet aggregation and plaque formation at the endothelial cell, predisposing a patient to vascular damage and occlusion and increasing the risk of mortality (Ramallal et al., 2017).

The results of our study demonstrated that there was a significant positive association between dietary acid load and risk of CVD mortality, so that by increasing one score in dietary acid load, the rate of CVD mortality increased by 2%. In line with the present study, *Shamima* et al. (Akter et al., 2017) detected a strong relationship among PRAL, NEAP scores, and CVD mortality rates. *Minseon* et al. (Park et al., 2015) also linked an increased dietary acid load and a higher risk of CVD and all‐cause mortality. Previous research have shown that an increase in consumption of meat and its products, eggs, cheese, refined grains, and fish, and a decrease in consumption of vegetables and fruits lead to an increase in the dietary acid load and risk of chronic disorders, including cardiovascular diseases, hypertension, type 2 diabetes, and their mortality due to these diseases (Akter et al., 2017; Park et al., 2015.) In contrast the consumption of potassium bicarbonate, magnesium, fiber, vitamin C, calcium, and phytochemicals, which exist abundantly in fruits and vegetables (as part of a healthy diet); are probably associated with decreased acid load of diet and a lower risk of CVD disorders. (Akter et al., 2017; Fatahi & Azadbakht, 2019; Han et al., 2016) Western dietary pattern contains abundant cheese and meat (acidogenic foods) and is deficient in vegetables and fruits (alkalizing foods) which increase the acid load of the dietary intake. (Kahleova et al., 2021) In addition, previous studies have also shown that a vegan dietary pattern is related to reducing dietary acid load. (Kahleova et al., 2021; Müller et al., 2021) Partial examination shows that a rise in acid load in diet, which is associated with an increase in blood acid levels, leads to the excretion of sodium in the body and an increase in cortisol secretion and insulin resistance. In this regard, the serum level of potassium reduces due to increased potassium excretion, which leads to an increase in hypertension by affecting vasodilation. (Adrogué & Madias, 2007) On the other hand, increased cortisol levels are related to metabolic syndrome and increased cardiovascular diseases and mortality risk. (Carnauba et al., 2017; Hur et al., 2015) Insulin resistance is also caused by a reduced tendency for insulin to bind to its receptor in an acidic environment, which is directly related to the risk of CVD and all‐cause mortality. (Ormazabal et al., 2018; Zhang et al., 2017)

Phillips et al. (2019) and Fung et al. (2008) showed that when adherence to the DASH diet increases, the risk of cardiovascular diseases and mortality decreases. This decrease is due to the consumption of more vegetables, fruits, nuts and seeds, whole grains, and low‐fat dairy products and restrictions on the consumption of red meat, sugars, sweets, beverages, and total and saturated fat. The mechanism of impact of this diet can be attributed to its compounds such as potassium, sodium, magnesium, calcium, fiber, and antioxidants. In this diet, high fiber intake leads to reduction in the levels of LDL, cholesterol, triglycerides, hypertension, CRP, and the risk of obesity and overweight, and improves insulin sensitivity and endothelial function (Levitan et al., 2013). DASH diet with emphasis on magnesium and potassium sources such as dark‐green leafy vegetables, seeds, nuts, and whole grains can play a role in controlling hypertension and stroke. Magnesium and potassium reduce inflammatory cytokines and increase nitric oxide levels, thereby affecting the severity of heart diseases and mortality due to them (Dokken, 2008; Rifai et al., 2015). According to our results, it was observed that adherence to the DASH diet in cardiovascular patients could reduce the risk of CVD mortality by 20.4%; however, this decrease was not statistically significant. In line with our study, (Aigner et al., 2018) did not find a strong relationship between DASH score and mortality from stroke.

In the present study, we did not find a significant correlation between AHEI score and CVD‐related deaths. Contrary to our study, *Emily* et al. (Hu et al., 2020) reported that higher AHEI scores led to lower risk of cardiovascular diseases and mortality by 16% and 34%, respectively. The AHEI score can assess the quality of the diet and predict the risk of death due to chronic diseases. The index measures the consumption of whole grains, fruits, vegetables, fish, dairy, processed red meat, and alcohol. Also, (Akbaraly et al., 2011) in their study found that higher AHEI score had a significant inverse relationship with CVD mortality, while similar to the current study, no significant association was observed between this score and cancer‐related mortality. The most important reason for the inconsistency of the results of our study with other studies can be the short follow‐up time.

In their study over 4 years, *Emily* et al. (Levitan et al., 2013) recorded 1385 mortality cases from HF. In their study, similar to ours, there was no statistically significant relationship between MDS score and mortality. While (Dokken, 2008; Hodge et al., 2018) found that the more the adherence to the Mediterranean diet, the lower the risk of mortality at a young age. The Mediterranean diet improves systolic hypertension and lowers CRP, fibrinogen, oxidative stress, and serum cholesterol, thereby reducing the incidence of chronic disorders and their mortality.

One of the main weaknesses of this study was the short follow‐up time. Also, in this study, recall diet was not examined for nutritional assessment and only FFQ was used. Another weakness of this study was the recording of nutritional data in the baseline state and no other dietary record was recorded during the follow‐up period; as a result, we were unable to assess the patients' dietary changes. Using self‐reporting for food evaluation can also increase overestimation and underestimation. In this study, we failed to assess important factors such as disease severity, type of treatment during illness, and evaluation of health care that are associated with mortality. One of the strengths of this study is the study of dietary indices instead of evaluating specific micronutrients in the controls and inhibition of cardiovascular disease and its mortality. Also, in this study, four important indices were examined simultaneously, and this is of great importance. To summarize and comment definitively in this regard, it is suggested that studies with more follow‐up years and the use of cohort studies should be carried out in other regions simultaneously.

## 5 CONCLUSION

The current study indicated that increasing dietary acid load and the use of inflammatory food compounds increase the risk of CVD mortality. Adherence to the DASH diet can also be associated with decreased risk of mortality due to cardiovascular diseases in patients. Therefore, paying attention to the dietary acid load and acid balance along with the use of diets with antiinflammatory properties and focusing on the DASH diet in CVD patients may be effective in preventing CVD mortality. However, further studies are needed in this regard.

## CONFLICT OF INTEREST

None declare.

## Data Availability

The data supporting the finding of this study are available from the corresponding author upon reasonable request.
